# A Comprehensive Investigation of Infill Geometry Effects on the Mechanical Performance of Polymer 3D Printed Components

**DOI:** 10.3390/polym18010111

**Published:** 2025-12-30

**Authors:** Faisal J. Alzahrani, Yasser S. Alzahrani, Mohammed T. Alamoudi, Mojahed Alkhateeb

**Affiliations:** 1 Department of Mechanical Engineering, Faculty of Engineering-Rabigh, King Abdulaziz University, Jeddah 21589, Saudi Arabia; 2 Department of Mechanical Engineering, Faculty of Engineering, King Abdulaziz University, Jeddah 21589, Saudi Arabia; ysalzahrani1@kau.edu.sa; 3 Advanced Material Technologies Institute, Energy and Industry Sector, King Abdulaziz City for Science and Technology, Riyadh 11442, Saudi Arabia; malamoudi@kacst.gov.sa; 4 Department of Industrial Engineering, Faculty of Engineering-Rabigh, King Abdulaziz University, Jeddah 21589, Saudi Arabia; mmalkhateeb@kau.edu.sa

**Keywords:** polymer additive manufacturing, printing pattern, tensile test

## Abstract

Fused filament fabrication (FFF, often called FDM) is widely used in polymer additive manufacturing; however, it suffers from mechanical anisotropy and weak bonding in the Z direction. This work examines how the infill pattern influences the tensile response of PLA parts at fixed printing conditions. Dog-bone specimens (PLA, four patterns: grid, honeycomb, rectilinear, adaptive cubic) were printed and tested in tension (n = 3 per pattern). Grid yielded the highest ultimate tensile strength, whereas honeycomb produced the largest Young’s modulus; rectilinear was intermediate and adaptive cubic was trailed in both metrics. X-ray diffraction of printed PLA showed a broad halo at 16–20° (2θ) with weak α-form reflections, consistent with largely amorphous microstructure after FFF. Together, the results indicate that, at constant material and nominal infill, pattern selection alone can shift the strength–stiffness balance, with grid favoring strength and honeycomb favoring stiffness.

## 1. Introduction

Additive manufacturing (AM) has experienced substantial advancements in recent decades, facilitating its integration into a wide array of industrial applications [1]. Within AM technologies, material extrusion—commonly known as fused filament fabrication (FFF) or Fused Deposition Modeling (FDM)—is extensively utilized due to its affordability, operational simplicity, and compatibility with various thermoplastic polymers, including PLA, ABS, PC, and TPU [2]. In FDM, a heated nozzle deposits molten filament layer by layer, enabling the fabrication of complex geometries with reduced material waste and minimal post-processing relative to conventional manufacturing methods.

Extensive research has investigated the impact of FFF processing parameters on mechanical performance, including layer thickness, infill density, infill pattern, printing speed, and extrusion temperature [3]. Previous studies have indicated that increasing infill density typically enhances tensile and flexural strength, while layer thickness and extrusion temperature influence interlayer bonding quality and failure mechanisms [4,5,6]. Elevated extrusion temperatures and optimized print speeds improve mechanical properties up to an optimal point, after which degradation may occur due to thermal instability or extrusion defects [7,8].

From a design standpoint, the infill pattern is among the most practical and cost-neutral variables available to engineers, enabling strength and stiffness optimization without altering material or process settings. Recent investigations underscore the significant influence of infill pattern geometry on stiffness, strength, and surface characteristics. Kadhum et al. [9] identified substantial variations in tensile response across different infill geometries and polymer types, while Agrawal et al. reported pronounced coupled effects among infill pattern, density, and layer height [10]. Additional studies have examined pattern scaling [11], three-dimensional infill architectures [12], and the effects of rotational symmetry [13], further demonstrating the sensitivity of mechanical response to internal geometry.

Despite these advancements, mechanical anisotropy continues to limit FFF components, with reduced strength commonly observed along the build (z) direction due to weak interlayer bonding. Interlayer adhesion is determined by a sequence of contact, neck formation, molecular diffusion, and chain re-entanglement processes [14], all of which are highly sensitive to thermal history and cooling rate [15,16,17]. Recent approaches, such as selectively increasing infill temperature while maintaining standard contour settings, have demonstrated potential for enhancing tensile strength without sacrificing dimensional accuracy [18].

Several studies have explored the optimization of printing parameters to enhance interlayer bonding and reduce anisotropy. Moradi et al. studied the tensile strength for six types of infill patterns of horizontally printed PLA samples. They concluded that the highest strength and Young’s modulus was achieved by the triangle pattern, while wiggle and fast honeycomb showed the highest elongation and absorbed energy [19]. El Magri et al. identified layer thickness as the most significant parameter affecting Z-direction tensile strength of Acrylic Styrene Acrylate (ASA) components, with optimal performance achieved at 0.155 mm layer height and elevated nozzle temperatures [20]. Sánchez et al. examined large-format additive manufacturing and showed that fiber reinforcement unexpectedly reduced tensile and flexural strength in ASA composites when printed in both x- and z-orientations [21]. Vanaei et al. reported that maintaining the previous layer’s temperature just below crystallization temperature increased PLA tensile strength by approximately 23% [22]. Sedighi et al. measured reductions of 60% and 45% in tensile and flexural strength, respectively, for vertically vs. horizontally printed PC specimens, reinforcing the severity of build-direction dependence [23]. More recent research further highlights geometric effects. Cañero-Nieto et al. demonstrated that infill pattern alters the effective cross-sectional area at the gauge length, thereby shifting tensile strength and failure modes in PLA parts [24], while Li et al. showed that honeycomb pattern and grid pattern provide different bending and tensile performance trade-offs in PLA beams [25].

Recent reviews have identified the lack of systematic pattern-level understanding as a significant gap in material extrusion research [26]. Therefore, the present work examines the interlayer bonding strength in the Z-direction prints by evaluating the tensile performance of different infill patterns (grid, honeycomb, rectilinear, and adaptive cubic) of PLA under fixed material, nominal infill density, and processing. Also, mechanical response is correlated with near-filament microstructural features using X-ray diffraction to clarify the process–structure–property relationship. The results provide practical guidance for selecting infill strategies that balance stiffness and strength, thereby enhancing the reliability of FFF components in load-bearing applications.

## 2. Materials and Methods

Commercial polylactic acid (PLA) filament (ELEGOO, 1.75 mm) has a density of 1.25 g/cm^3^, and a glass temperature of 60 °C was used. Tensile specimens followed relevant ASTM geometries: dog-bones per ASTM D638 [27]. CAD models were built in SolidWorks, 2022 SP04. Nominal dimensions were as follows: tensile gauge length 165 mm, width 19 mm, thickness 7 mm, flexural bar length 127 mm, width 12.7 mm, thickness 3.2 mm.

All specimens were printed on a fused filament fabrication printer (Creality K1, nozzle 0.4 mm; Creality 3D Technology Co., Ltd., Shenzhen, China) with fixed parameters across groups and infill pattern was the experimental factor (rectilinear, honeycomb, adaptive cubic, and grid), as shown in Table 1:

For Layer Height Settings, the layer height was set to the standard 0.2 mm, a balanced setting for a 0.4 mm nozzle that offers a good compromise between detail and speed. For the first layers, increase the height to improve adhesion to the build plate, which is crucial for print success. For Shell and Wall Settings, a high number of perimeters in wall loops maximizes part strength and rigidity. Using three wall loops and 5 top/bottom layers creates a thick, solid exterior that is highly resistant to external forces. This is a standard configuration for functional prototypes or end-use parts that will be subject to mechanical stress. Furthermore, the 60% infill density is the most significant indicator of a strength-focused print. While 10–20% is typical for general use, 60% ensures that the internal volume is substantially filled with material, drastically increasing the part’s compressive and tensile strength and showing sustainable testing results.

The speed parameters were meticulously selected to balance the need for efficient sample fabrication with the requirement for high-quality, consistent test specimens. The 30 mm/s first-layer speed is a critical control measure that maximizes bed adhesion and ensures a consistent, reliable foundation for all samples, thereby eliminating a significant source of initial print failure and dimensional variation. For the main body of the print, a high speed of 300 mm/s was maintained for the internal features (inner walls and infill) to minimize overall fabrication time. Crucially, the outer wall speed was deliberately reduced to 200 mm/s. This slower rate is essential to ensure optimal dimensional accuracy and a consistent surface finish on the exterior of the test samples.

These parameters represent a methodologically controlled configuration for the fabrication of high-integrity test specimens. The combination of a high 60% infill density and a robust 3-loop wall structure was explicitly chosen to maximize the mechanical strength of the samples, ensuring they meet the requirements for rigorous testing protocols. Furthermore, the differential speed strategy was implemented to ensure dimensional consistency and surface quality, critical factors for minimizing experimental variability and ensuring the reliability and repeatability of subsequent mechanical characterization.

**Figure 1:**
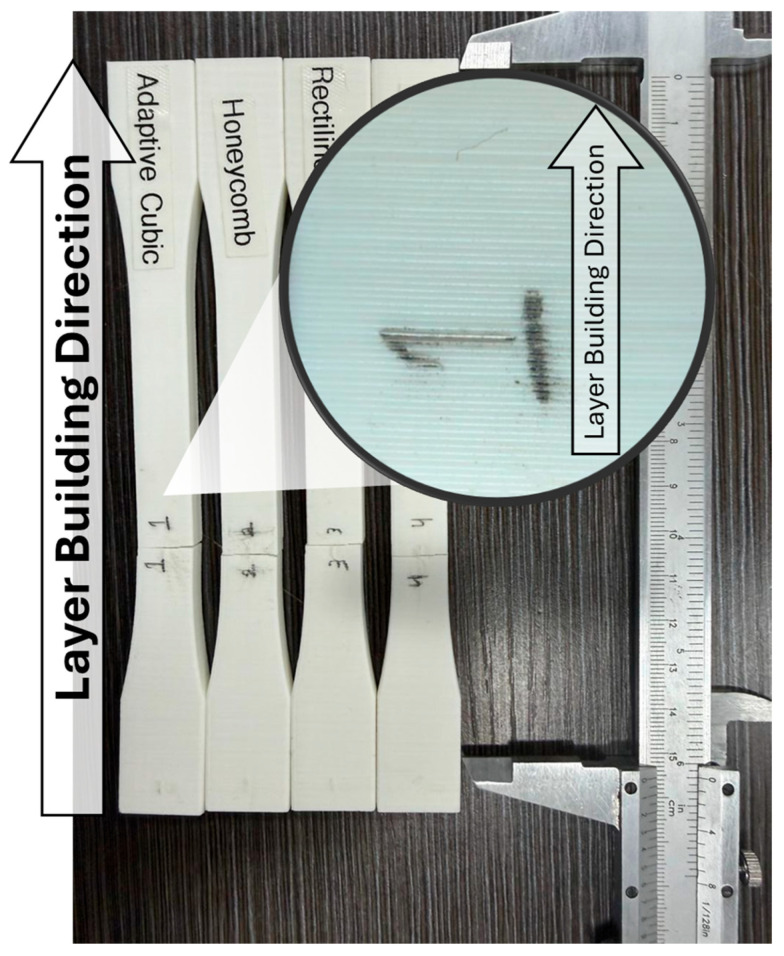
Samples’ details.

Tensile tests were conducted on a universal testing machine. Each sample was properly gripped and aligned during testing, and real-time measurements of force, displacement, and stress–strain response were recorded for subsequent analysis in OriginPro2024. For each condition, three replicates were tested. From the tensile curves, we obtained ultimate tensile strength (UTS), 0.2% offset yield strength, and Young’s modulus to examine how patterning influenced fracture. Unlike most previous studies, in which tensile specimens are typically printed with layers oriented parallel to the loading direction, the present work focuses on specimens fabricated with the layer-building direction aligned with the vertical axis, coinciding with the long dimension of the dog-bone specimen. This configuration enables a direct assessment of tensile behavior governed by interlayer bonding, which represents the weakest direction in fused filament fabrication. Figure 1 shows the sample’s failure location, layer-building direction, and four different infill patterns.

X-ray diffraction (XRD) analysis was performed to examine the phase composition of the printed PLA. X-ray diffraction (XRD) measurements were performed using a Rigaku MiniFlex 600 diffractometer (Rigaku Corporation, Tokyo, Japan) controlled by MiniFlex Guidance software (version 3.0.3.6), and data analysis was conducted using PDXL 2 software (version 2.8.1.1, Rigaku Corporation). The system operated at a voltage of 40 kV and a current of 15 mA. Scans were recorded over a 2-theta range of 5° to 100°.

Simultaneous thermal analysis (STA) was performed using a NETZSCH STA 449 F3 Jupiter system (NETZSCH-Gerätebau GmbH, Selb, Germany). The sample was heated from 30 °C to 400 °C at 10 K/min under a nitrogen atmosphere. Data acquisition and analysis were carried out using NETZSCH Proteus software (Version 6.1).

## 3. Results and Discussions

PLA specimens printed with four infill patterns (grid, honeycomb, rectilinear, adaptive cubic) were tested in tension under identical conditions (dog-bone geometry, 1 mm min^−1^, 6 × 12 mm section; n = 3 per pattern). Stress and strain were calculated from the data as follows:

(1) $$\sigma =\frac{F}{A_{0}},\;\;\;\;\;\;A_{0}=72\;{mm}^{2}$$

(2) $$\varepsilon =\frac{∆L}{L_{e}},\;\;\;\;\;\;L_{e}=50\;mm$$

**Figure 2:**
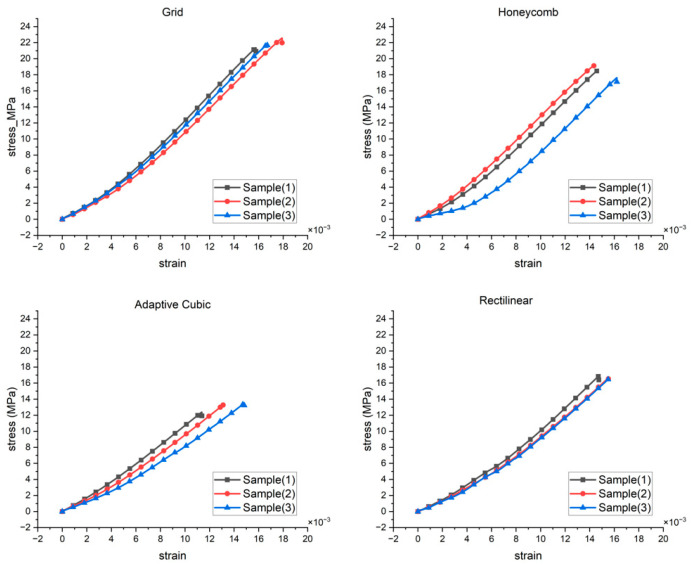
Comparative visualization of the tensile strength of all different patterns.

The failure of the printed specimens was primarily governed by layer-to-layer separation, as indicated by the abrupt stress drop at the ultimate tensile strength. Figure 2 shows a comparative visualization of tensile strength for the different infill patterns. Ultimate tensile strength (UTS) was determined as the maximum stress from the tensile stress–strain curves of 3D-printed specimens manufactured using different infill patterns. Values are reported as individual test results and mean ± standard deviation (n = 3). The ultimate tensile strength (UTS) of the 3D-printed specimens was strongly influenced by the infill geometry, as summarized in Table 2. Among the investigated configurations, the Grid infill exhibited the highest tensile strength (21.91 ± 0.61 MPa), followed by Honeycomb (18.42 ± 0.77 MPa), Rectilinear (16.65 ± 0.20 MPa), and Adaptive Cubic (13.05 ± 0.58 MPa).

The superior UTS observed for the Grid infill can be attributed to the presence of continuous and aligned load-bearing paths along the tensile loading direction. This configuration promotes efficient stress transfer between adjacent filaments and layers, delaying the onset of fracture. Such behavior has been widely reported for grid- and line-based infill patterns under uniaxial tensile loading, where filament alignment plays a dominant role in strength rather than stiffness.

In contrast, the Adaptive Cubic infill exhibited the lowest tensile strength despite showing relatively high elastic stiffness. This highlights a critical distinction between elastic response and ultimate failure behavior in additively manufactured polymers. While Adaptive Cubic structures distribute elastic loads effectively, their complex three-dimensional geometry may introduce stress concentrations and weaker interlayer bonding regions, which act as preferential sites for crack initiation under tensile loading.

These mechanical rankings are consistent with established cellular-architecture mechanics: continuous axial load paths in lattice-like grids favor peak stress capacity, while hexagonal connectivity efficiently carries elastic bending and shear, raising the effective modulus at a given relative density. Idealized honeycomb models predict that the in-plane modulus scales with wall slenderness and connectivity, explaining why a honeycomb can be stiffer than a grid even when the mean strut size is smaller [28].

The standard deviation of UTS values provides insight into the repeatability of the tensile failure process. Rectilinear infill exhibited the least variability (±0.20 MPa), suggesting stable, repeatable failure mechanisms across specimens. Conversely, Honeycomb infill showed higher scatter, reflecting sensitivity to local geometric imperfections, cell-wall continuity, and interlayer adhesion quality.

These observations indicate that infill patterns with higher geometric complexity may offer improved stiffness or energy absorption, but at the expense of increased variability in tensile strength. This trade-off should be carefully considered when selecting infill strategies for load-bearing components fabricated by fused filament fabrication.

From a design perspective, the results demonstrate that Grid infill is the most effective configuration for maximizing tensile strength, whereas rectilinear infill provides superior repeatability with moderate strength. Honeycomb infill offers a balance between strength and structural efficiency. In contrast, Adaptive Cubic infill may be better suited for applications where stiffness or multi-directional load distribution is prioritized over ultimate tensile capacity.

These findings emphasize that tensile strength optimization in 3D-printed polymers requires careful consideration of infill geometry, as failure behavior is governed not only by material properties but also by the internal architecture and the quality of filament bonding.

**Figure 3:**
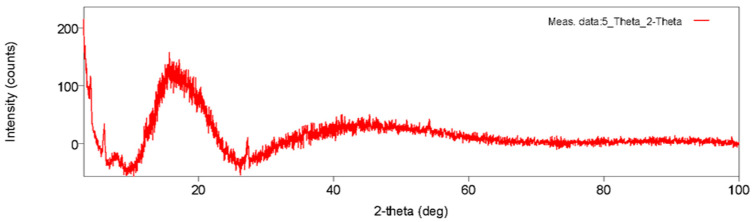
X-ray diffraction of printed PLA showing amorphous halo (16–20° 2θ) with weak α-PLA reflections.

X-ray diffraction (Figure 3) shows a broad halo centered at 16–20° (2θ) with weak reflections indexed to the (110)/(200) family of α-PLA, indicating a predominantly amorphous microstructure with limited crystallinity after printing; typical for material extrusion builds owing to rapid cooling and layerwise thermal cycling. This assignment and interpretation match prior reports that place the dominant α-PLA (110)/(200) near ~16.7° (2θ) [29,30].

**Figure 4:**
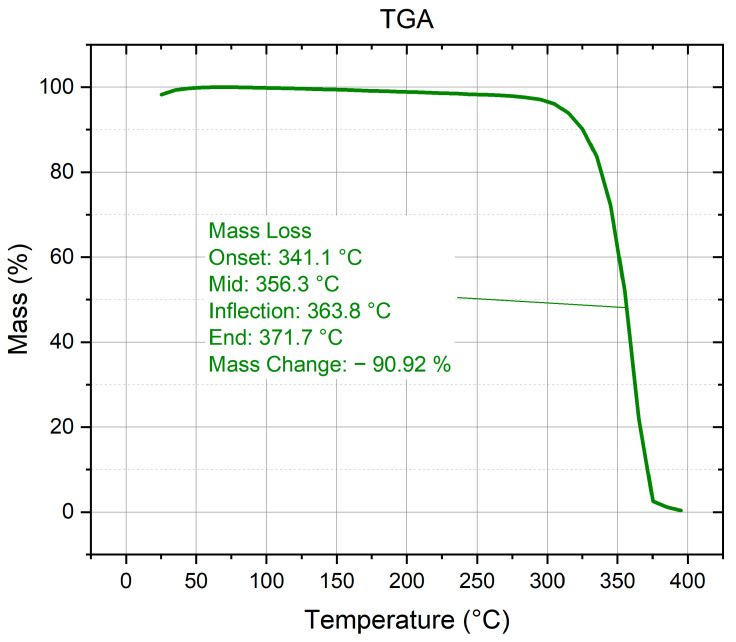
Thermogravimetric analysis of printed PLA.

Thermogravimetric analysis (Figure 4) shows a single main mass loss window from ≈341–371 °C with ~98.5% total loss, consistent with the known decomposition range of neat PLA (onset ≳ 290–300 °C; peak rate typically within ~350–380 °C, depending on grade and heating rate) [31].

**Figure 5:**
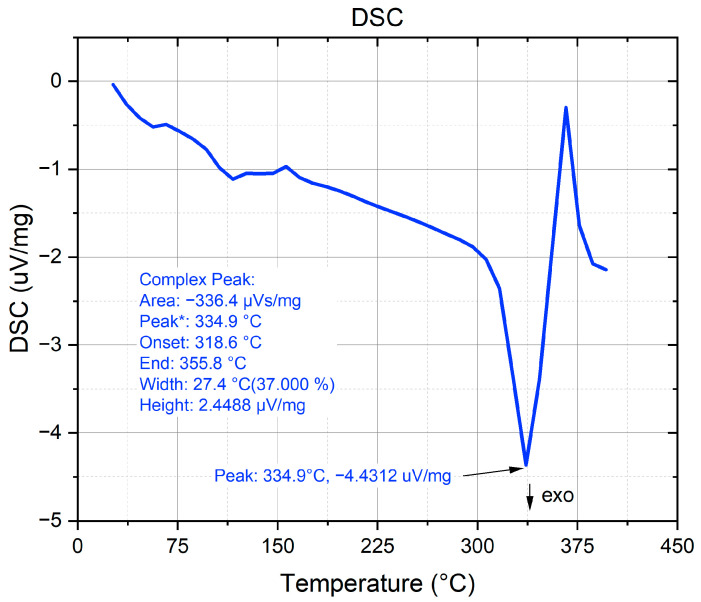
Differential scanning calorimetry of printed PLA: melting endotherm (onset/peak/end = 159.2/168.3/179.9 °C).

Differential scanning calorimetry (Figure 5) exhibits a single, well-defined melting endotherm with onset/peak/end at 159.2/168.3/179.9 °C. This temperature window is characteristic of the melting of α-form PLA crystallites, for which peaks centered near ≈165–180 °C are widely reported for neat grades at conventional heating rates. The peak temperature (~168 °C) represents the most probable lamellar thickness, while the endotherm breadth reflects a realistic distribution of lamellar thicknesses, i.e., both features are consistent with the Gibbs–Thomson relation linking crystal thickness to melting temperature in semi-crystalline polymers. The high-temperature endset (~180 °C) indicates a minor population of thicker or more perfected lamellae that persist to higher temperatures before fusion. Overall, the measured melting interval aligns with reference data for commercial PLA resins and with prior accounts of α-phase melting behavior.

Optical measurements of infill features (reported as “infill thickness,” i.e., a characteristic strut/bead dimension) differentiate the patterns (Table 3 and Table 4). Mean ± SD (µm) and coefficient of variation CV% are as follows: adaptive cubic 212 ± 24 (11.3%), grid 465 ± 84 (18.1%), honeycomb 327 ± 82 (25.2%), and rectilinear 329 ± 53 (16.1%). The grid’s larger load-bearing features align with its higher UTS in Table 1. The honeycomb achieves the highest modulus despite a smaller mean feature size than grid, reinforcing that topology/connectivity, not size alone, controls stiffness in cellular architectures.

The pattern property shows a clear strength–stiffness trade-off at fixed nominal settings: grid maximizes UTS and extension to break, whereas honeycomb maximizes elastic modulus; rectilinear is intermediate and adaptive cubic trails. Mechanistically, this aligns with mesostructural load–path arguments. Grid provides continuous, axially aligned struts that carry tensile loads with less bending, which favors higher peak stress capacity and greater extension before fracture. Honeycomb, by contrast, distributes load through closed-cell connectivity that elevates the effective elastic response through wall bending and shear, raising E at the same nominal density, even when the mean strut size is smaller than in the grid. This behavior is consistent with cellular solids scaling, where in-plane modulus scales strongly with wall slenderness/connectivity [2,32].

The geometric statistics of the infill further support this interpretation. The optical measurements show larger characteristic strut dimensions for the grid (mean ≈ 465 µm) than for the honeycomb/rectilinear (≈327–329 µm) and adaptive cubic (≈212 µm). The larger load-bearing section in grid tracks has a higher UTS, whereas honeycomb achieves the top modulus despite a smaller mean strut size, reinforcing that topology and connectivity, not size alone, govern stiffness in these lattices. It is worth noting that these variations in strut thickness arise from the specific toolpath generation for each pattern (e.g., overlapping intersections in Grid), despite using a constant nozzle diameter.

The microstructure–mechanics linkage is also coherent across the measurements. XRD indicates a predominantly amorphous PLA with weak α-form reflections in the 16–20° (2θ) window, which is typical for material extrusion builds due to rapid thermal cycling. In that regime, topology and weld quality tend to dominate the elastic/strength response more than crystalline fraction, so it is unsurprising that pattern choice alone moves both UTS and E at constant infill. The DSC melting interval (onset/peak/end ≈ 159/168/180 °C) is consistent with α-lamellae of realistic thickness distribution; the breadth of the endotherm reflects lamellar heterogeneity and is expected for printed PLA [2,6,22].

The results also reflect interface-limited failure typical of FFF/MEX parts. Interlayer welds originate from neck formation and inter-diffusion between adjacent filaments; the time–temperature history of each layer governs how far the interface progresses along the neck-growth/diffusion path before cooling arrests it. Under tensile loading, failure often initiates at welds/voids where local stress constraint is highest. Patterns that create longer stretching-dominated load paths (grid) reduce bending in struts and move the critical section away from the weld; patterns with more bending-dominated walls (honeycomb) elevate stiffness yet retain moderate UTS because local stresses still concentrate at weld toes. This thought is consistent with melt-bonding theory and prior reports on anisotropy and bonding quality in material extrusion polymers [6,15,17,20,33].

From a design perspective, the present ranking suggests simple selection rules at constant material and nominal infill; grid works when peak tensile capacity and ductility are limiting (e.g., tie-bars, clips, snap-fits loaded along the strut direction) and honeycomb when elastic stiffness is limiting (e.g., panels, covers, fixtures constrained by deflection). If mass or throughput constraints favor adaptive cubic, expect a trade-off in both UTS and E relative to grid/honeycomb. For engineers integrating these infills into parts, two practical checks improve reproducibility: (i) confirm relative density equivalence across patterns by weighing specimens (mass-normalized comparisons remove minor slicer-dependent differences in wall/perimeter deposition) and (ii) report raster angles and perimeter counts, as they influence load-path continuity and weld density [2,6,33].

## 4. Conclusions

This study shows that, at fixed material and nominal infill, the infill pattern alone can be used to tune the tensile response of printed PLA. Among the four patterns examined, grid delivered the highest tensile capacity and the largest extension to break; honeycomb maximized the elastic modulus; rectilinear was intermediate in both metrics; and adaptive cubic trailed in both strength and stiffness. XRD indicated a predominantly amorphous microstructure with weak α-PLA reflections; DSC exhibited a single melting endotherm (onset/peak/end ≈ 159.2/168.3/179.9 °C), and TGA showed a main decomposition window near 341–371 °C, all consistent with the thermal history of material extrusion builds.

The results provide practical selection rules for design at constant density: choose a grid when peak tensile capacity and ductility are limiting (e.g., load-bearing struts or clips loaded along the filament path), and choose a honeycomb when stiffness/deflection is the constraint (e.g., panels and covers). The optical measurements support this interpretation: larger characteristic struts in the grid align with higher UTS, whereas honeycomb’s closed-cell connectivity elevates modulus even with smaller mean strut size, underscoring the role of topology and load-path continuity rather than feature size alone.

From a manufacturing perspective, the results suggest that infill patterns exhibiting stable mechanical performance and low variability are more suitable for large-scale production. Infill designs such as Grid and Rectilinear offer a favorable balance of tensile strength, stiffness, and repeatability, making them attractive candidates for mass production via fused filament fabrication. Furthermore, controlling build orientation and process parameters is essential to ensure consistent quality in industrial-scale applications.

## 5. Limitations and Future Work

Three limitations frame the scope: (i) the present n = 3 per condition limits statistical power; and (ii) only one parameter set and orientation were studied. (iii) This study focuses on the tensile behavior of 3D-printed specimens with different infill patterns. While tensile properties provide fundamental insight into the mechanical performance of additively manufactured polymers, other mechanical characteristics such as fatigue resistance, impact strength, and fracture toughness were not investigated within the scope of the present work.

Follow-on work will examine how infill pattern and printing parameters, such as printing speed and layer height, jointly affect the tensile behavior of 3D-printed components. In future work, the number of replicates will be increased (n ≥ 5), the mean ± SD will be reported, and ANOVA will be used for pattern × orientation. Normalization by relative density (mass) will be used to compare specific properties, and fractography will be added to document failure at welds/voids. Mapping a small process matrix (infill %, raster angles, nozzle temperature) and extending to additional lattice topologies (e.g., gyroid, triangular) will help identify robust operating regions where pattern choice is decisively superior and generalizable across printers and slicers.

## Figures and Tables

**Table 1:** Three-dimensional-printed samples processing parameters.

Parameter	Value	Unit	Parameter	Value	Unit
Lyer height	0.2	mm	First layer	30	mm/s
First-layer height	0.3	mm	First-layer infill	60	mm/s
Wall loops	3		Outer wall	200	mm/s
Top shell layers	5	layers	Inner wall	300	mm/s
Top shell thickness	0.8	mm	Travel speed	500	mm/s
Top surface density	100	%			

**Table 2:** Tensile properties of PLA by infill pattern (grid, honeycomb, rectilinear, adaptive cubic).

Infill Type	Sample	UTS (MPa)
Grid	Grid 1	21.37
	Grid 2	22.57
	Grid 3	21.78
	Mean ± SD	21.91 ± 0.61
Honeycomb	Honeycomb 1	18.47
	Honeycomb 2	19.17
	Honeycomb 3	17.63
	Mean ± SD	18.42 ± 0.77
Adaptive Cubic	Adaptive Cubic 1	12.39
	Adaptive Cubic 2	13.27
	Adaptive Cubic 3	13.48
	Mean ± SD	13.05 ± 0.58
Rectilinear	Rectilinear 1	16.86
	Rectilinear 2	16.63
	Rectilinear 3	16.46
	Mean ± SD	16.65 ± 0.20

**Table 3:** Raw infill strut/bead thickness measurements by pattern (µm).

#	Adaptive Cubic (µm)	Grid (µm)	Honeycomb (µm)	Rectilinear (µm)
1	179.611	515.749	406.113	409.321
2	203.421	530.978	446.965	331.959
3	224.891	382.941	380.615	366.274
4	206.495	524.137	268.812	354.781
5	243.393	363.588	273.715	350.910
6	—	366.087	257.402	342.948
7	—	400.525	243.343	405.007
8	—	570.782	253.251	296.284
9	—	529.879	—	269.236
10	—	411.942	—	254.700
11	—	—	—	257.449
12	—	—	—	306.111

**Table 4:** Summary statistics of infill strut/bead thickness by pattern: mean and standard deviation (µm).

Pattern	Mean Length (µm)	Standard Deviation (µm)
Adaptive Cubic	211.5622	23.99839
Grid	464.9629	84.24943
Honeycomb	326.9064	82.34571
Rectilinear	328.7483	52.89023

## Data Availability

The data presented in this study are available on request from the corresponding author due to legal reasons.
